# Publisher Correction: Dynamics for El Niño-La Niña asymmetry constrain equatorial-Pacific warming pattern

**DOI:** 10.1038/s41467-020-18650-y

**Published:** 2020-09-11

**Authors:** Michiya Hayashi, Fei-Fei Jin, Malte F. Stuecker

**Affiliations:** 1 Department of Atmospheric Sciences, SOEST, University of Hawai‘i at Mānoa, 2525 Correa Rd., Honolulu, HI 96822 USA; 2 Department of Oceanography and International Pacific Research Center, SOEST, University of Hawai‘i at Mānoa, 1680 East-West Rd., Honolulu, HI 96822 USA; 3 Present Address: Center for Global Environmental Research, National Institute for Environmental Studies, 16-2 Onogawa, Tsukuba, 305-8506 Ibaraki Japan

**Keywords:** Atmospheric dynamics, Climate and Earth system modelling, Projection and prediction, Physical oceanography

Correction to: *Nature Communications* 10.1038/s41467-020-17983-y, published online 28 August 2020.

The original version of this Article contained errors in the author affiliations.

Affiliations 1 and 2 incorrectly read ‘Department of Atmospheric Sciences, SOEST, University of Hawaiian ʻOkina at Mānoa, 2525 Correa Rd., Honolulu, HI 96822, USA’ and ‘Department of Oceanography and International Pacific Research Center, SOEST, University of Hawaiian ʻOkina at Mānoa, 1680 East-West Rd., Honolulu, HI 96822, USA’ instead of the correct ‘Department of Atmospheric Sciences, SOEST, University of Hawai‘i at Mānoa, 2525 Correa Rd., Honolulu, HI 96822, USA’ and ‘Department of Oceanography and International Pacific Research Center, SOEST, University of Hawai’i at Mānoa, 1680 East-West Rd., Honolulu, HI 96822, USA.’

This has now been corrected in both the PDF and HTML versions of the Article.

