# Supplementary material for: Dynamics for El Niño-La Niña asymmetry constrain equatorial-Pacific warming pattern
Source: Nat Commun. 2020 Aug 28;11:4230. doi: 10.1038/s41467-020-17983-y (PMC7455730; doi:10.1038/s41467-020-17983-y)
Supplement: Supplementary file 1 — Supplementary Information [file 41467_2020_17983_MOESM1_ESM.pdf]

Supplementary Information for

Dynamics for El Niño-La Niña asymmetry constrain equatorial-Pacific warming pattern

by Hayashi et al.

M. Hayashi*, F.-F. Jin*, & M. F. Stuecker

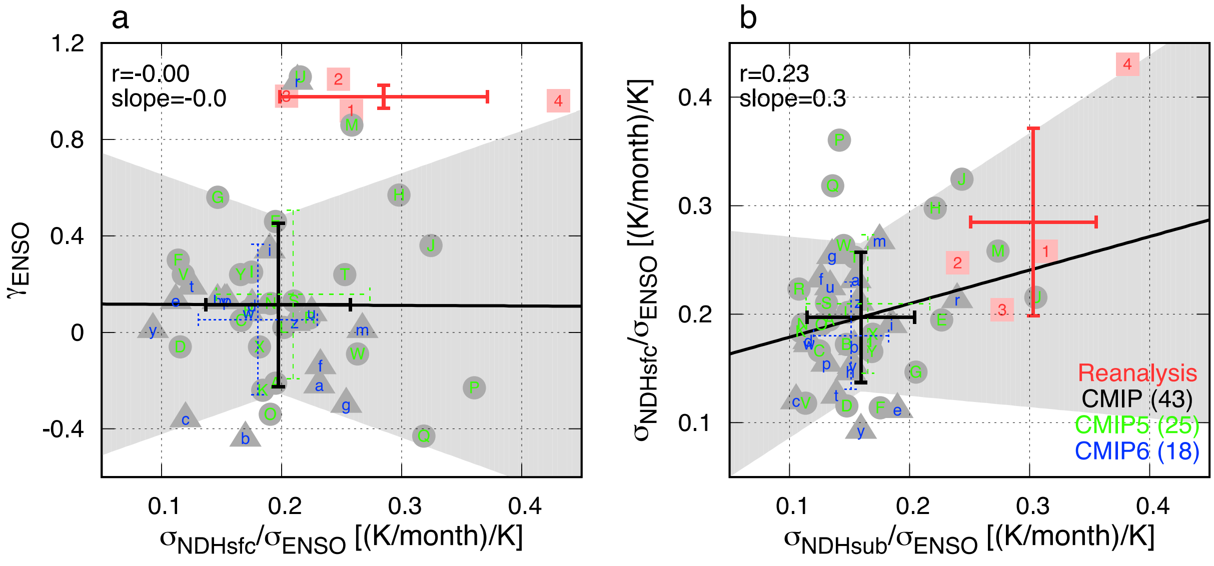

Supplementary Figure 1 | Role of the surface-layer NDH on ENSO asymmetry. a, Skewness of sea surface temperature (SST) anomalies in the Niño-3 region as functions of the efficiency of surface-layer nonlinear dynamical heating (NDH), which is defined as the NDH terms averaged in the upper 50 m depth at 100˚W–180˚ and 1˚S–1˚N. **b,** The relationship of the NDH efficiencies in the subsurface- and surface layers. Error bars denote the one standard deviation range for all models (black), CMIP5 (green with circles), CMIP6 (blue with triangles), and reanalysis (red with squares). In each bracket, the number of available models is indicated. The linear fitting lines for all the models are shown with shading for the 95% confidence ranges of slopes and intercepts.

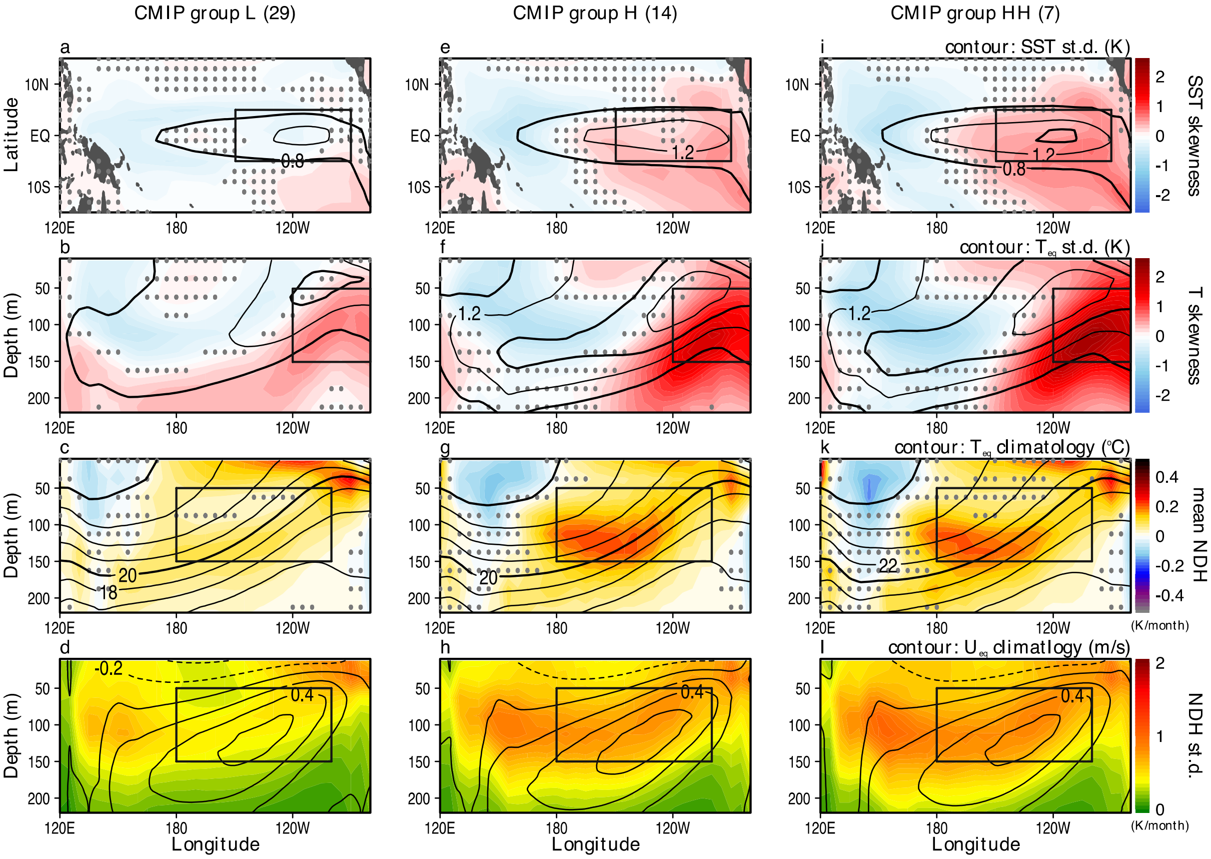

Supplementary Figure 2 | Simulated nonlinearity in the equatorial Pacific Ocean. Same as in Fig. 1, except for subgroups L, H, and HH of CMIP models. **a**, Horizontal map of the skewness γ (shading) and standard deviation σ (contours, K) of the sea surface temperature (SST) anomaly. **b**, Equatorial cross section of γ (shading) and σ (contours, K) of the potential temperature anomaly. **c**, The long-term mean of nonlinear dynamical heating (NDH) (shading, K month^{-1}) and potential temperature (contours, ˚C). **d**, σ of the NDH (shading, K month^{-1}) and the long-term mean of the zonal ocean current (contours, m s^{-1}). The historical simulations of subgroup L of CMIP models are used in **a**–**d**. Dots in **a**–**c** indicate the shading values that are not statistically significant at the 95% confidence level. Boxes represent the Niño-3 region in **a** while the averaging regions for the mean and σ of NDH in **c** and **d** and the skewness of subsurface temperature in **b**. **e–h,** Same as in **a**–**d**, except for subgroup H. **i–l,** Same as in **a**–**d**, except for subgroup HH

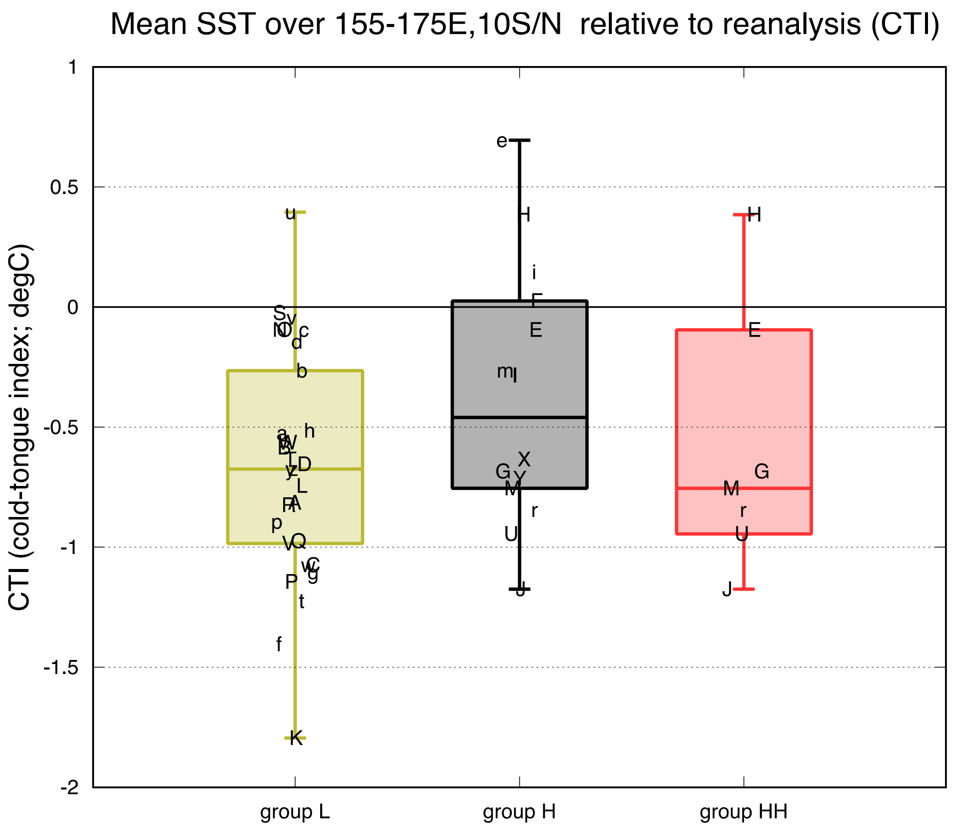

Supplementary Figure 3 | Mean-state cold-tongue bias in climate models. Inter-quartile ranges and maximum/minimum levels of cold-tongue index (CTI: climatological sea surface temperature bias averaged over 155˚–175˚E and 10˚S–10˚N; ref.1) in CMIP historical simulations (groups L, H, and HH). The bias is defined as the difference from the multi-reanalysis mean.

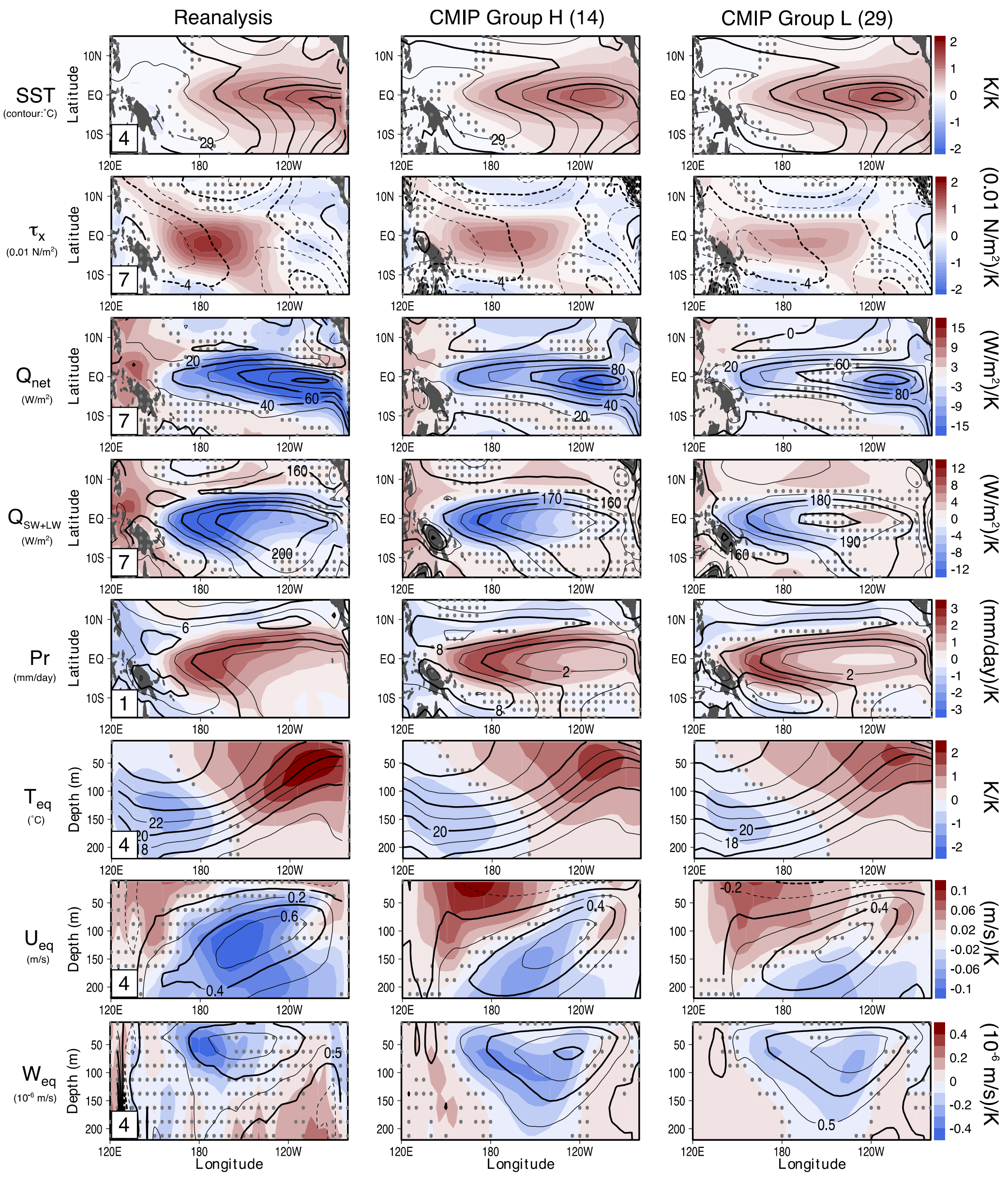

Supplementary Figure 4 | Normalized ENSO variability and mean state. Showing are the composites of the reanalysis products and CMIP historical simulations classified into groups H and L. The detrended anomalies are regressed to the detrended Niño-3 sea surface temperature (SST) anomalies (K). From the top to bottom, the variables for the regression (color) and long-term mean (contour) are SST, zonal wind stress, net surface heat flux, radiative surface heat flux, precipitation, potential temperature, zonal current, and vertical velocity. The depth-longitude cross sections are for equatorial averages from 1˚S–1˚N. The dotted regions denote the values are not statistically significant at the 95% confidence levels. The numbers of available datasets for reanalysis are shown in each panel while those for two CMIP groups at the top.

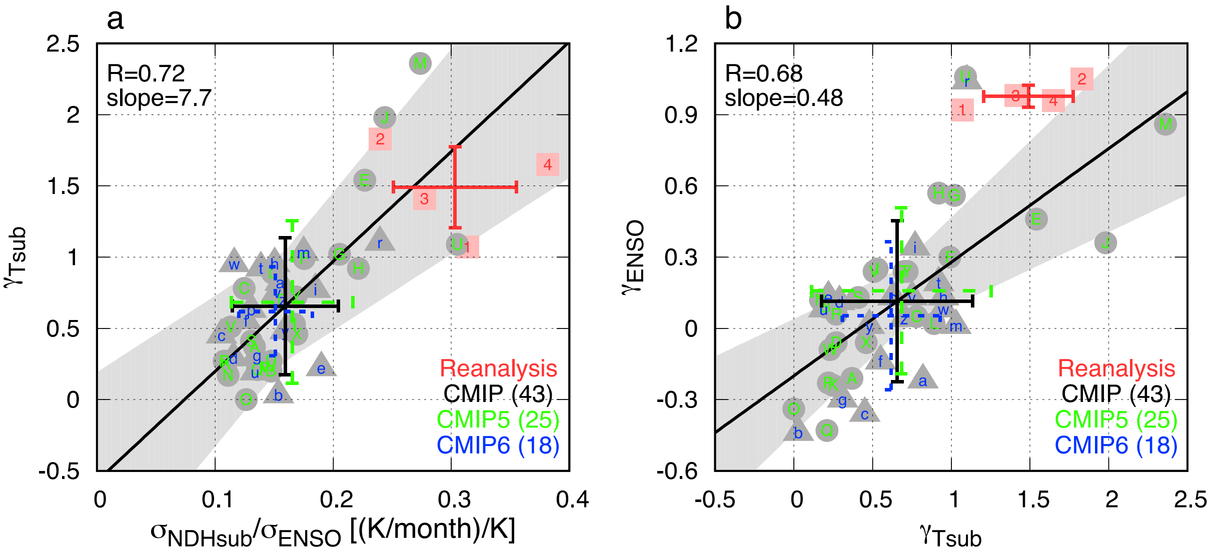

Supplementary Figure 5 | Skewness in the subsurface and surface temperature anomalies. **a,** Skewness of the eastern-Pacific equatorial temperature in the subsurface (γ_{Tsub}; 80˚–120˚W, 1˚S–1˚N, 50–150 m depth) as function of the efficiency of subsurface nonlinear dynamical heating. **b,** Skewness of the Niño-3 sea surface temperature anomalies (γ_{ENSO}; ENSO asymmetry) as a function of γ_{Tsub}. Error bars denote the one standard deviation range for all models (black), CMIP5 (green with circles), CMIP6 (blue with triangles), and reanalysis (red with squares). In each bracket, the number of available models is indicated. The linear fitting lines for all the models are shown with shading for the 95% confidence ranges of slopes and intercepts.

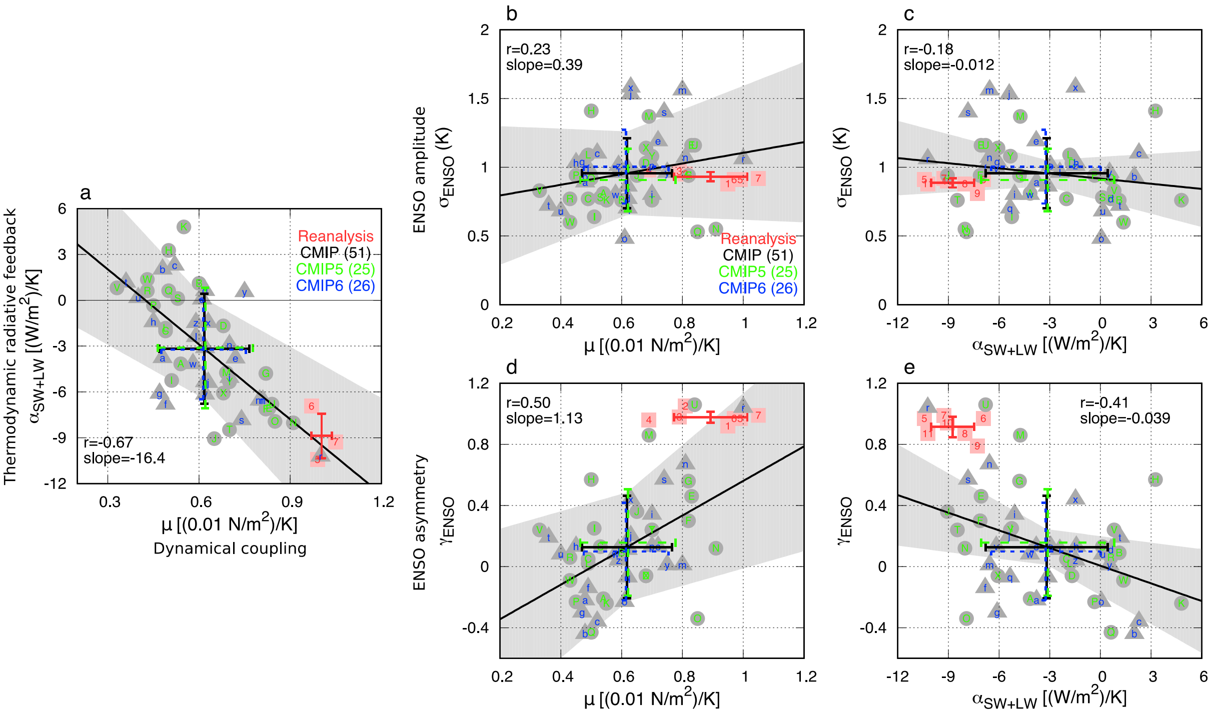

Supplementary Figure 6 | Dynamical coupling and thermodynamic radiative feedback. a, Regression coefficients of the detrended anomalies of the zonal wind stress (μ) in the central Pacific domain and the radiative surface heat flux in the Niño-3 and Niño-4 regions (α_{SW+LW}) onto the detrended Niño-3 sea surface temperature anomalies. **b–e,** ENSO's amplitude and asymmetry as functions of μ and α_{SW+LW}. The green upper cases over the triangles and blue lower cases over the circles indicate 25 CMIP5 and 26 CMIP6 models, respectively, and the red plots indicate multiple reanalysis datasets (Supplementary Table 1). Error bars denote the ranges of one standard deviation for all the models (black), CMIP5 (green), CMIP6 (blue), and reanalysis (red). The linear fittings for all the models are shown with the 95% statistical significance intervals of the slopes and intercepts.

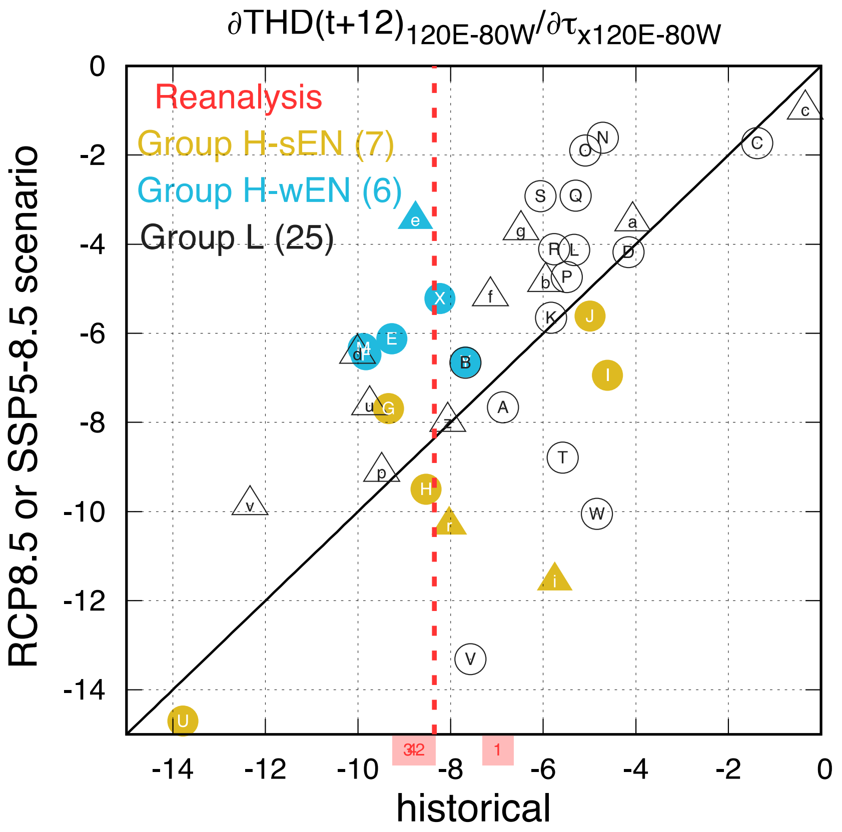

Supplementary Figure 7 | Future change of wind-thermocline coupling. Showing are the delayed thermocline responses to the anomalous zonal wind stress over the equatorial Pacific basin in the historical and RCP8.5 or SSP5-8.5 scenario simulations in 38 CMIP models in groups H and L. Orange and cyan marks indicate the strengthening (H-sEN) and weakening (H-wEN) of the ENSO amplitude in group H, respectively, and the opened marks represent the models in group L. The triangles are for CMIP6 and the circles are for CMIP5. The red vertical line indicates the average of the ocean reanalysis estimates (red squares on the x-axis). The thermocline depth (THD) is measured as the depth at the sharpest vertical gradient of potential temperature. Each plot represents the 12-month lagged regression of the detrended THD anomaly (m) to the detrended zonal wind-stress anomaly (0.01 N m^{-2}) in the equatorial Pacific domain (120˚E–80˚W, 5˚S–5˚N) for the whole period in the historical simulations and 2051–2100 in the RCP8.5 and SSP5-8.5 scenario simulations.

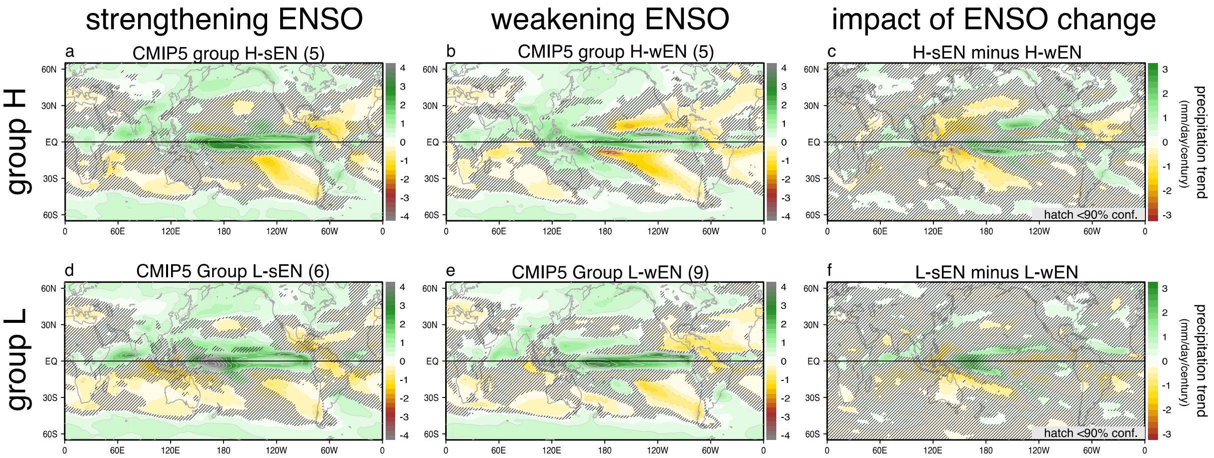

Supplementary Figure 8 | Precipitation trends due to ENSO amplitude change. a–c, Linear trends of the total precipitation in groups H-sEN and H-wEN and their difference (H-sEN minus H-wEN) in the CMIP5 Representative Concentration Pathway 8.5 scenario simulations. **d–f,** As in **a–c** but for groups L-sEN and L-wEN. Hatched areas over shading indicate that the values are not statistically significant at the 90% confidence level.

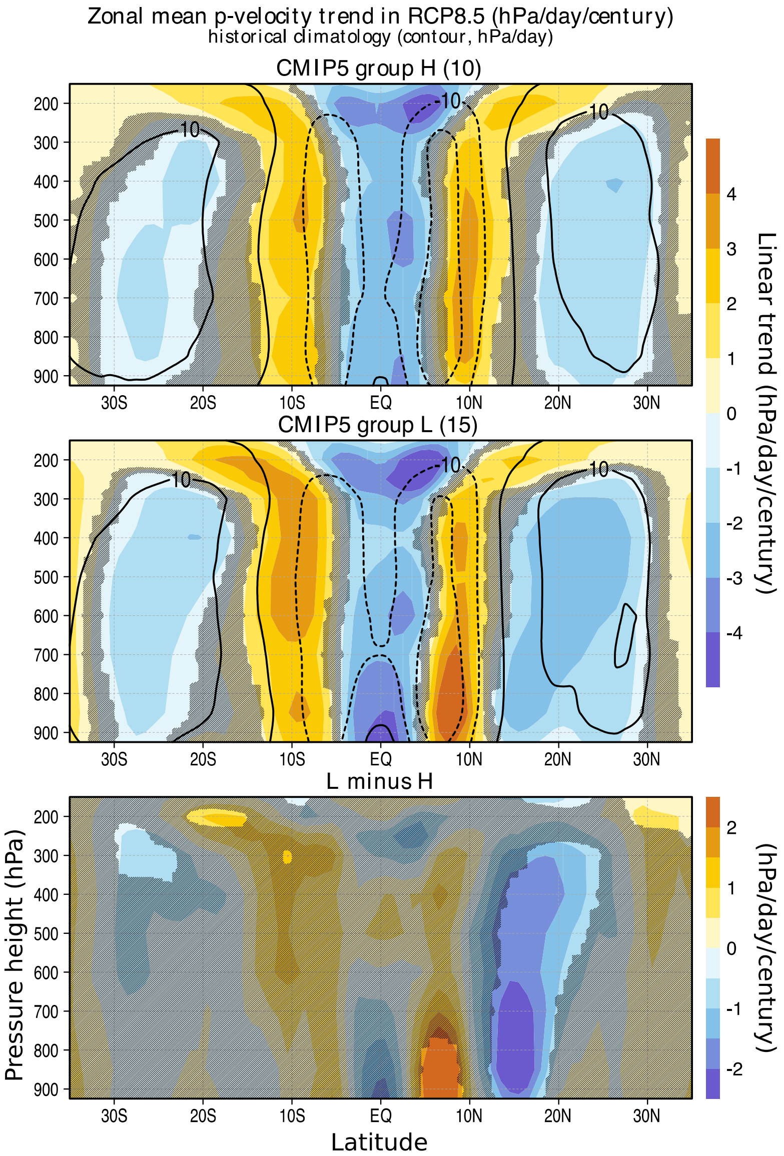

Supplementary Figure 9 | Hadley circulation change under global warming. Composites of the zonal-mean pressure velocity for the linear trends of the RCP8.5 simulations and the long-term mean of the historical simulations in 25 CMIP5 models. Difference between H and L is shown in the bottom panel. The hatched area indicates the values are not statistically significant at the 95% confidence level.

Supplementary Table 1 | Data availability for CMIP models and reanalysis products.

Symbol	Data name	Group	Historical, surface	Scenario, surface	Historical, ocean	Scenario, ocean
	CMIP5 models		Period: 1850–2005	Period: 2006–2100	Period: 1850–2005	Period: 2006–2100
A	ACCESS1-0	L				
B	ACCESS1-3	L				
C	bcc-csm1-1	L				
D	CanESM2	L				
E	CCSM4	H, HH				
F	CESM1-BGC	H				
G	CESM1-CAM5	H, HH				
H	CMCC-CESM	H, HH				
I	CMCC-CM	H				
J	CNRM-CM5	H, HH				
K	CSIRO-Mk3-6-0	L				
L	GFDL-CM3	L	1860–2005**		1860–2005**	
M	GFDL-ESM2M	H, HH	1861–2005**		1861–2005**	
N	GISS-E2-R	L				
O	GISS-E2-R-CC	L				
P	HadGEM2-CC	L	1860–2005**		1860–2005**	
Q	HadGEM2-ES	L	1860–2005**		1860–2005**	
R	IPSL-CM5A-LR	L				
S	IPSL-CM5A-MR	L				
T	IPSL-CM5B-LR	L				
U	MIROC5	H, HH				
V	MPI-ESM-LR	L				
W	MRI-CGCM3	L				
X	NorESM1-M	H				
Y	NorESM1-ME	H				
	CMIP6 models		Period: 1850–2014	Period: 2015–2100	Period: 1850–2014	Period: 2015–2100
a	ACCESS-CM2	L				
b	ACCESS-ESM1-5	L				
c	BCC-CSM2-MR	L				
d	CanESM5	L				
e	CESM2	H				
f	CNRM-CM6-1	L				
g	CNRM-ESM2-1	L				
h	E3SM-1-1	L		N/A*		N/A*
i	EC-Earth3-Veg	H				
j	FGOALS-f3-L	N/A*		N/A*	N/A*	N/A*
k	GFDL-CM4	N/A*			N/A*	N/A*
l	GFDL-ESM4	N/A*			N/A*	N/A*
m	GISS-E2-1-G	H		N/A*		N/A*
n	GISS-E2-1-H	N/A*		N/A*	N/A*	N/A*
o	INM-CM5-0	N/A*			N/A*	N/A*
p	IPSL-CM6A-LR	L				
q	KACE-1-0-G	N/A*			N/A*	N/A*
r	MIROC6	H, HH				
s	MIROC-ES2L	N/A*			N/A*	N/A*
t	MPI-ESM-1-2-HAM	L		N/A*		N/A*
u	MPI-ESM1-2-HR	L				
v	MRI-ESM2-0	L				
w	NESM3	L		N/A*		N/A*
x	NorESM2-MM	N/A*		N/A*	N/A*	N/A*
y	SAM0-UNICON	L		N/A*		N/A*
z	UKESM1-0-LL	L				
	Reanalysis/observations		Each data period			
1	ORAS3		1959–2011			
2	ORAS5		1979–2017			
3	SODA331		1980–2015			
4	GODAS		1981–2017			
5	ERA-Interim		1979–2018			
6	ERA5		1979–2018			
7	TropFlux		1979–2017			
8	OAFlux		1984–2009			
9	CERES		2001–2017			
10	SRB		1984–2007			
11	ISCCP-FH		1984–2009			
	GPCP		1979–2018			

*N/A: not available; **The data period is shown only when different from the default value.

Supplementary References

1. Grose, M. R. et al. Insights from CMIP6 for Australia's future climate. *Earth's Future* **8(5),** e2019EF001469 (2020).
